# The psychological syndrome associated with Long-COVID: A study protocol

**DOI:** 10.3389/fepid.2023.1193369

**Published:** 2023-08-22

**Authors:** Raquel Gómez Bravo, Alexandre Infanti, Joël Billieux, Mark Ritzen, Psy-Long-COVID Consortium, Steffen Mortiz, Claus Vögele, Charles Benoy

**Affiliations:** ^1^ Centre Hospitalier Neuropsychiatrique (CHNP), Rehaklinik, Ettelbruck, Luxembourg; ^2^ Department of Behavioural and Cognitive Sciences, Faculty of Humanities, Education, and Social Sciences, University of Luxembourg, Esch-sur-Alzette, Luxembourg; ^3^ Institute of Psychology, University of Lausanne (UNIL), Lausanne, Switzerland; ^4^ Universitäre Psychiatrische Kliniken Basel, Zentrum für Psychosomatik und Psychotherapie, Basel, Switzerland; ^5^ Department of Clinical Research, University of Basel, Basel, Switzerland

**Keywords:** Long-COVID, COVID-19, persistent COVID-19 symptoms, post-COVID-19 syndrome, mental health

## Abstract

**Introduction:**

Chronic post-viral syndromes, including Long-COVID, are characterized by a range of persistent symptoms that occur following a viral infection. Psychological symptoms are prevalent in Long-COVID patients and can have a significant impact on their quality of life. However, the specific patterns of psychological symptoms, their associations with physical symptoms, and the factors predicting their severity remain poorly understood.

**Aims:**

This study aims to explore and systematically assess psychological symptoms in Long-COVID, to identify syndrome clusters based on these symptoms, to examine their relationship with physical symptoms, and to investigate the influence of pandemic-related variables.

**Methods:**

Descriptive, cross-sectional study with data collected through an online questionnaire across several EU countries, from February 2020 to December 2022. Participants were recruited using public relations, the social media and information campaigns directed at the public and health professionals using snowball sampling.

**Results:**

The findings will allow to phenotype Long-COVID related psychological symptom clusters based on self-reports. facilitating improved assessment and treatment approaches.

**Conclusions:**

The results will provide important knowledge for the public health management of the public healh management of Long COVID. The findings will contribute to a better understanding of the psychological symptoms associated with Long-COVID and the development of specific treatment guidelines for psychological burden associated with Long-COVID, thereby supporting management strategies to combat the after-effects of the COVID-19 pandemic, enhancing their overall well-being and quality of life.

## Introduction

Chronic post-viral syndromes refer to a range of symptoms that persist for a prolonged period after a person has recovered from a viral infection. These symptoms can include physical, psychological, and neuropsychological symptoms, which are common and have a significant impact on individuals’ quality of life, ability to work or attend school, and their mental health. For example, chronic Post-SARS (Severe Acute Respiratory Syndrome) that emerged from South East Asia in early 2003, was characterized by persistent fatigue, diffuse myalgia, weakness, depression and non-restorative sleep (1). These symptoms can vary widely, involving either one or more persistent COVID-19 related symptoms, or even new symptoms, and occurring weeks and months after the infection. They have been described under the terms Long-COVID or Post-COVID (2).

Long-COVID may result in long-term disability or chronic illness. A considerable proportion of Long-COVID patients report persistent distress and impairment in their global level of functioning (3). In a Swedish study (initially investigating long-term immunity after mild COVID-19), it was found that even low risk individuals with mild COVID-19 reported a range of long-term symptoms that disrupted work, social and home life (4).

The prevalence of Long-COVID-syndrome varies widely depending on the population studied, the length of time used to define persistence of symptoms and study design. In a recent study published in January 2023, it has been estimated that 50%–70% of hospitalized cases and 10%–30% of COVID-19 non-hospitalized patients experience long-term symptoms (2, 5–7). Further research is needed to better understand not only the prevalence, risk factors, and long-term outcomes of Long COVID, but also the underlying mechanisms to develop effective treatments. Several post-COVID syndrome categories have been described, including multisystemic physical, persistent psychological and neuropsychological symptoms [e.g., (3, 8, 9)].

Even though the available evidence has methodological limitations, a recent systematic review and a meta-analysis suggest that fatigue and sleep disturbance are the most prevalent symptoms in Long-COVID syndrome (10, 11). Other frequently reported symptoms include anxiety, depression or other mental health conditions, headaches, cognitive impairment (also known as “brain fog”), loss of concentration, and muscle aches/myalgia (3, 4, 8, 12–14). In a retrospective cohort study of 273.618 survivors of COVID-19, Taquet et al. (15) found that anxiety and depression were the most common features of Long-COVID. They also observed higher incidences of cognitive symptoms in the elderly and in hospitalized patients or intensive treatment unit (ITU) patients. The prevalence of persistent symptoms was higher after COVID 19 compared to influenza, suggesting that the infection with SARS-CoV-2 might be involved (15).

Some studies suggest similarities between the clinical presentation of Long-COVID syndrome and the better-known Myalgic Encephalomyelitis/Chronic Fatigue syndrome (ME/CFS). It is important to note that Long-COVID is a newly recognized condition, and the underlying mechanisms are still not well understood. It remains unclear, therefore, whether they are the result of direct neurological damage from the virus, immune system dysregulation or other factors and their long-term implications. Current evidence seems to suggest that both viral and host factors may play a role. For example, viral factors such as persistent viral replication or ongoing immune activation may contribute to ongoing symptoms. Host factors such as genetics, age, and pre-existing medical conditions may also influence an individual's susceptibility to developing chronic post-viral symptoms. In addition, there are some differences in the clinical presentation of Long-COVID and ME/CFS, and the two conditions may have different underlying causes (2). Nevertheless, the similarities between the two conditions suggest that there may be some overlap in the mechanisms that lead to chronic post-viral syndromes (16, 17).

As described in the previous section, the current evidence shows a high number of long-COVID patients with psychological symptoms [e.g., (15)]. Although little is known about the risk factors for persistent psychological symptoms associated with Long-COVID, the currently most widely accepted factors include the severity of COVID-19, female sex, a history of mental disorders, the presence of comorbidities, and elevated levels of inflammatory markers. In a similar vein, the pathophysiological mechanisms underlying Long-COVID remain only partially explained, whereas the role of other factors for the development of Long-COVID, for example those associated with the treatment of COVID-19 (e.g., ITU admission), has not been investigated yet. The potential impact of indirect effects of the COVID-19 pandemic such as uncertainty, social isolation, financial constraints, and health recovery on Long-COVID has been highlighted but also remains largely unexplored (18, 19).

In summary, (1) a considerable proportion of individuals who are diagnosed with COVID-19 report a Long-COVID syndrome, (2) this syndrome includes a range of physical, psychological and neuropsychological symptoms, (3) the incidence of psychological symptoms in Long-COVID is high, and (4) these syndromes have not yet been sufficiently and adequately assessed.

In general, it is well established that many patients with physical diseases also experience psychological symptoms or even have a mental disorder comorbid with the physical condition (20). This may be (1) a direct result of pathophysiological processes associated with the physical condition, (2) be indirectly linked as an indirect consequence of the challenging situation of being diagnosed with a physical condition, (3) be a result of medical interventions to treat the physical condition, or (4) present an interaction of any of these (21). The present study aims to identify patterns of psychological symptoms that are specific for Long-COVID, thus contributing to better assessment and treatment.

## Objectives

The overall research goals concern the exploration and systematic assessment of psychological symptoms of Long-COVID and the factors associated with it.

Specific objectives are:

1) To describe different syndromes associated with psychological symptoms in relation with Long-COVID and generate different groups based on their psychological symptoms (anxiety, depression and somatoform disorders)
2) To investigate whether these syndrome clusters differ in relation to physical symptoms.
3) To examine whether syndrome clusters show differences in terms of other pandemic-related variables, including psychosocial and medical factors.

Data on the psychological symptoms of Long-COVID were collected using an online survey from February 2020 to December 2022. The project will allow for the retrospective assessment of predictive factors, which are associated with more severe mental health symptoms linked to a Post Covid syndrome (e.g., pre-existing mental disorders, social context, other treatments, blood type).

## Materials and methods

### Design

This study involves an online, descriptive, cross-sectional survey including 184 items. Participation was voluntary, anonymous and could be withdrawn at any time. The questionnaire was available in German and French. Its completion took 30 min on average.

Questions asked covered a broad range of psychological symptoms (e.g., depression, overall anxiety, specific anxiety, fatigue, impact of health on life quality, perceived stress, traumatic experiences, somatic symptoms experiences) and person- and pandemic-related factors (e.g., sociodemographic factors, vaccination status, time of infection, acute phase, acute treatment setting, health-related factors, impact of the pandemic on several domains of personal life). To control for the effects of pre-existing conditions, we included a comprehensive assessment of possibly confounding variables in the survey. In addition, a range of possible risk factors that may have chronologically preceded the Coronavirus infection were also collected (Table 1).

**Table 1:** Variable groups collected.

Person-related variables:	Assessment of psychological symptoms and complaints:
• Age, gender, weight, height	• General anxiety: General Anxiety Disorder-7 (GAD-7), 7 Items
• Country of residence, educational level, civil status, family situation, household, children	• Depression: Patient Health Questionnaire (PHQ-9), 10 Items
• Occupational status, professional area, shift work, Home-Office	• Physical complaints: Somatic Symptoms Experiences Questionnaire (SSEQ), 15 Items)
• Previous somatic/physical diseases	• Fatigue: Fatigue Assessment Scale (FAS), 10 Items
• Actual somatic/physical disease	• Psychological Stress: Perceived Stress Scale (PSS), 10 Items
• Previous mental illness	• Traumatic experiences: Trauma Screening Questionnaire (TSQ), 10 Items
• Current mental illness	
• General list of symptoms and complaints	
• General questionnaire on COVID-19 exposure and impact	
• Other health-related questions (e.g., COVID-vaccination status, time of infection, date of acute COVID infection, medication, smoking, substance use, pre-existing illness, current treatment, blood type, etc.)	

### Recruitment and informed consent

Participants were recruited through public relations and information campaigns directed at the public and to health professionals. In addition, we collaborated with several Long-COVID units, intensive care units, infectious diseases departments and mental health care departments (e.g., psychiatry departments), so that individuals with Long-COVID were directly referred to the present study. This study is part of a multi-center and international project, and the procedure was the same in all participating institutions.

#### Participants

##### Sample size

Snowball sampling was used to conduct this research.

##### Inclusion criteria

Any volunteer was welcome to participate in this project. For the present study, respondents were selected according to the WHO definition of Long COVID: those experiencing new symptoms 3 months after the initial SARS-CoV-2 infection, with these symptoms lasting for at least 2 months without other explanation (22).

##### Data analysis

Data analysis and interpretation of the results will be carried out from January to April 2023.

We will compute a cluster analysis following the recommendations provided by Hair et al. (23), which consist of combining hierarchical and non-hierarchical approaches. For the cluster generation, we will use the total scores of the GAD-7, PHQ-4 (PHQ-9 excluding items which refer to physical symptoms), and all the SEEQ dimensions. These variables will be scaled and centered for the analyses. Before running the cluster analyses, it will be ensured that no collinearity between these variables exists, and the distribution the scales verified. Hierarchical clustering will provide us with a suggested number of clusters using the Ward method with squared Euclidian distances. To obtain a suggested number of clusters, the *NbClust* R package (24) will be used. In this package, several indices are computed (kl, ch, hartigan, ccc, scott, marriot, trcovw, tracew, friedman, rubin, cindex, db, silhouette, duda, pseudot2, beale, ratkowsky, ball, ptbiserial, frey, mcclain, dunn, hubert, sdindex, dindex, sdbw). For each index, a suggested number of clusters will be obtained, the most outstanding suggested number of clusters is then highlighted and recommended for further steps (non-hierarchical clustering). Once the suggested number of clusters is obtained, we will assess its relevance for our main assumptions to decide the final number of clusters to generate. Then, the generation of the clusters will be followed by non-hierarchical K-means cluster analysis. As an alternative solution, a supervised machine learning model called Affinity Propagation will be computed. This model is relevant since it represents a completely data driven approach method. Unlike other clustering algorithms, such as K-means, Affinity Propagation does not require the number of clusters to be pre-defined. Instead, the algorithm automatically determines the number of clusters from a data driven perspective. The algorithm works by iteratively exchanging messages between data points, which convey information about how well one data point can serve as an exemplar for another data point. The messages are used to update the availability and responsibility matrices, which represents the confidence that a data point has in its potential exemplar and the confidence that an exemplar has in its responsibility to represent a particular data point. During each iteration, the availability and responsibility matrices are updated until they converge to a stable solution. The exemplars for the data points are determined by finding the data points with the highest net responsibility. The results of both cluster generation approaches will then be compared regarding their scores on the PHQ-4 (without physical symptoms), GAD-7, and SSEQ dimensions. The more relevant cluster solution will be chosen for further analyses.

To compare the clusters regarding physical symptoms, we will compute a contingency table and report Chi-Squared tests, Cramer's V and effect sizes. Moreover, we will compute *post hoc* analyses using the *chisq.post hoc.test* R package (25) which uses Bonferroni correction, and is based on residuals of Pearson's Chi-squared Test for Count Data.

The same procedure will be applied to examine whether syndrome clusters show differences in terms of other pandemic-related variables, including psychosocial and medical factors. If the external variables are continuous, a Kruskal–Wallis test will be computed with Dunn's test as *post hoc* analysis with Bonferroni correction.

### Ethics and dissemination

- Ethical approval

The study was conducted according to the guidelines laid down in the Declaration of Helsinki. Ethical approval was not required as non-identifiable data was collected anonymously. This was confirmed by the respective responsible institutions [for example Ethical Commission of the Max-Planck-Institut für Psychiatrie, Munich, Germany, the Ethics Committee for Northwest and Central Switzerland (EKNZ) and the Ministry of Health Luxembourg].

- Participants’ information and consent

Online informed consent was requested from all participants before starting the questionnaire, including the right to withdraw from the study at any point. Volunteers did not receive any incentive for their participation.

If participants wished to be informed of the results of the project and relevant publications, they could contact the principal investigator through the contact provided in the online survey.

At the end of the study, participants could create a personal and completely anonymous code. If they wished to be informed of their own results, participants could receive these by sending this code to an email address, explicitly requesting this information (total scores of the questionnaires as well as an explanation of the interpretation scheme of the results).

- Data protection

Data was collected anonymously through the UNIPARK online software (www.unipark.com), taking into ccount the EU GDPR. The online survey was implemented via the following link: https://ww3.unipark.de/uc/long-covid/. IP addresses were not collected, and the data gathered did not allow for the identification of respondents.

Upon completion of data collection, data was downloaded and stored on a centrally managed server at the University of Luxembourg (UL). Afterwards, the data was deleted from the UNIPARK software. Data was stored in an AES encrypted ZIP archive to ensure its confidentiality and following the principles of privacy. The password is only known to CV and CB, the owners of the project file, and is not shared with any third party. If requested, access will be provided by the IP department upon authorization and data will be available for re-analysis during this period, following the Open Science policy for transparency, exchange, reproducibility, and accountability. The ZIP archive will be kept for 10 years in the shared drive and destroyed after this period, following the ethics guidelines of the University of Luxembourg.

## Results

The results will allow to phenotype Long-COVID related symptom clusters based on a broad range of self-reported psychological symptoms in a large international sample. These clusters based on psychological symptoms associated with Long-COVID may provide insight into the heterogeneity of psychological presentations among affected individuals. Additionally, the study aims to examine the relationship between psychological syndrome clusters and physical symptoms, shedding light on the interplay between physical and psychological aspects of Long-COVID. Furthermore, the associations between syndrome clusters and pandemic-related variables will provide valuable information regarding the impact of psychosocial and medical factors on Long-COVID outcomes. Finally, the results will help to identify different clusters of Long-COVID patients and support the design of tailored interventions.

Findings will be disseminated at scientific conferences after April 2023, by peer reviewed publications and different social media channels to reach both levels, the public and professionals.

## Discussion

Most of the extant literature only reports results of studies considering psychological symptoms among others, without specifically focusing on them. To date it is unclear whether psychological symptoms in the context of COVID-19 and Long-COVID are directly or indirectly related to the viral infection. Nevertheless, the range of physical symptoms is so broad, that it is unlikely that there is only one indirect negative effect of Long COVID on mental health, but several, potentially amplified by other factors [such as factors pertaining to the pandemic-related experience of social isolation, increased stress etc.; (2, 15, 26, 27)].

### Possible important findings of this research

We aim to identify clusters of psychological symptoms associated with Long-COVID and determine their prevalence. Moreover, we intend to investigate whether the identified syndrome clusters differ in relation to physical symptoms. Understanding the relationship between physical and psychological symptoms in Long-COVID can shed light on the interplay between these aspects and potentially uncover common underlying mechanisms.

The research includes the retrospective assessment of predictive factors associated with more severe mental health symptoms in Long-COVID, which may include pre-existing mental disorders, social context, other treatments, and blood type. Identifying predictive factors can help in identifying individuals at higher risk for developing severe psychological symptoms and inform targeted interventions. We include the investigation of pandemic-related variables, to examine the question, if syndrome clusters differ in terms of other pandemic-related variables, including psychosocial and medical factors. This broader assessment can provide a comprehensive understanding of the contextual factors that influence the development and manifestation of psychological symptoms in Long-COVID.

The results of the present study will provide important knowledge for public health management. The findings can contribute to better assessment and treatment of psychological symptoms in Long-COVID, inform the development of targeted interventions, enhance our understanding of the complex interactions between physical and psychological aspects of the condition and inform strategies to combat the after-effects of the COVID-19 pandemic.

## Limitations

The main limitations of this study are related to the use of the online questionnaire and online psychological and neuropsychological assessments that can be subject to various biases.

Online assessments are typically completed by individuals who have Internet access and feel comfortable using technology, which may exclude certain populations, such as those with limited Internet access or older individuals who may not have a sufficient level of eHealth literacy. Moreover, participation is voluntary, which introduces the potential for self-selection bias. Individuals who choose to participate may have specific motivations or characteristics that differ from those who opt out. This selection bias can impact the generalizability of the findings. Furthermore, the use of snowball sampling in this study can lead to biased sample selection, limiting the study's generalizability. Nevertheless, due to the pandemic situation, other recruitment methods such as random or convenience sampling were not feasible. Therefore, the study's findings should be interpreted with caution, and further research is needed. The referral process could also introduce biases as participants may refer others who share common characteristics. In addition, online assessments may not fully account for cultural or linguistic differences. Items and response options may not be applicable or adequately translated for diverse populations, leading to biased or inaccurate results across different cultural contexts.

Online questionnaires rely on self-reporting, which is also prone to response bias as participants may provide socially desirable responses, exaggerate, or downplay certain aspects of their behavior or symptoms. This bias can distort the accuracy of the assessment results as well. In addition, slow Internet connections or compatibility problems with specific devices or browsers, can introduce biases in the data and participants experiencing this kind of technical issues may rush through the assessment or abandon it altogether, leading to incomplete or skewed data.

The physical absence of a researcher or clinician who can observe and interpret non-verbal cues and behavior may affect the accuracy and depth of the assessment, as some conditions or symptoms may not be adequately captured through self-reporting alone. The lack of follow up in the online assessments might limit the validity of the results, compared to traditional assessments conducted by clinicians, where follow-up questions can help clarify ambiguous or inconsistent responses.

We acknowledge these drawbacks when interpreting the results of our study and recognize the limitations in capturing the complexity of psychological and neuropsychological phenomena. Nevertheless, the advantages of an online assessment are significant and therefore, important to consider. Online access to a survey increases the accessibility for participants and provides a standardized approach to assessing psychological and neuropsychological factors in long COVID patients, ensuring consistency in the assessment process, making it easier to compare results across patients and track changes over time. Long COVID is a complex condition that can have fluctuating symptoms and online questionnaires and assessments can be used to track their progress and identify changes in symptoms, allowing timely evaluation of these symptoms. This enables healthcare professionals to better understand the impact of long COVID on patients’ mental health and cognitive functioning, and to adjust treatment plans accordingly. Remote support can be provided in the future based on these assessments, through guidance, education, and treatment recommendations, improving access to care for individuals who may have limited access to specialized healthcare facilities. In addition, online assessments give long COVID patients an active role in their healthcare by supporting shared decision-making with their healthcare providers. Patient empowerment can play a crucial role in the management and recovery of long COVID patients.

## Conclusions

This study aims to contribute to a better understanding of the psychological symptoms associated with Long-COVID and their related factors. By identifying syndrome clusters and investigating their relationships with physical symptoms and pandemic-related variables, this research can provide valuable insight for improved assessment and treatment approaches for individuals with Long-COVID. Ultimately, these findings may help enhance the overall management and support provided to those affected by this chronic post-viral syndrome.

## Group members of Psy-Long-COVID Consortium

Steffen Mortiz: Universitätsklinikum Hamburg-Eppendorf, Germany. Angelika Erhardt: Max-Planck-Institut für Psychiatrie, Munich, Germany. Thérèse Staub: Centre Hospitalier du Luxembourg. Christian Huber and Undine Lang: Universitäre Psychiatrische Kliniken Basel, Switzerland; University of Basel, Switzerland. Steffi Weidt: Psychiatrische Universitätsklinik Zurich, Switzerland; University of Zurich, Switzerland. Kira Wolff and Juliane Müller: Universitätsklinikum Frankfurt am Main, Germany.
